# Intraabdominal and ganglionic desmoplastic small round cell tumor: a case series

**DOI:** 10.1186/s13256-021-03094-9

**Published:** 2021-10-12

**Authors:** S. Slim, I. Zemni, A. Bouida, M. Bouhani, N. Boujelbene, K. Mrad, R. Chargui, K. Rahal

**Affiliations:** 1 Surgical Oncology Department, Salah Azaiez Institute, Tunis, Tunisia; 2 Faculty of Medicine of Tunis, University of Tunis El Manar, Tunis, Tunisia; 3 Laboratory of Microorganisms and Active Biomolecules, Sciences Faculty of Tunis, Tunis, Tunisia; 4 Pathology Department, Salah Azaiez Institute, Tunis, Tunisia

**Keywords:** Desmoplastic small round cell, Chemotherapy, Surgery, Radiotherapy, Case report

## Abstract

**Introduction:**

Desmoplastic small round cell tumor is a rare malignancy with poor prognosis, affecting young male patients. It frequently presents as a large abdominal mass with widespread peritoneal involvement at diagnosis. In late stages, metastases may be present.

**Aim:**

We retrospectively reviewed patient characteristics, presenting symptoms, tumor pathology, treatment, and outcome of four patients with desmoplastic small round cell tumor at our institution.

**Cases presentation:**

The first three cases reported are 32-, 17-, and 30-year-old North African males with intraabdominal desmoplastic small round cell tumor treated by surgery, chemotherapy, and radiation therapy with different follow-ups. The final case is a 16-year-old North African male with ganglionic desmoplastic small round cell tumor but no evidence of a tissue mass. He underwent two lines of chemotherapy with no response. The patient was lost after 2 years of follow-up. In all cases, desmoplastic small round cell tumor was confirmed by presence of t(11,22) (p13,q12) translocation.

**Conclusion:**

Treatment of desmoplastic small round cell tumor is based on multidisciplinary therapy. Despite high-dose chemotherapy, extensive surgical resection, and radiotherapy, desmoplastic small round cell tumor remains lethal.

## Introduction

Desmoplastic small round cell tumor (DSRCT) is a rare and aggressive mesenchymal malignancy, recently described by Gerald and Rosai [1], characterized by a predilection for young males. Detection of the specific chromosomal translocation t(11;22)(p13;q12) confirms the diagnosis. This translocation, which results in active fusion protein involving the Ewing sarcoma (EWS) and Wilms tumor (WT1) genes, is pathognomonic [2, 3]. Serosal surfaces are most often affected, especially the peritoneal cavity. Due to its diffuse nature, DSRCT is frequently diagnosed at advanced stage. The prognosis of this disease is relatively poor, with high mortality.

Despite multimodal treatment based on chemotherapy, surgery, and radiation therapy, durable remission remains rare.

We report herein four cases of DSRCT.

## Case presentations

### Case 1

A 32-year-old North African man with no past medical history was referred by his attending physician to our department to manage an intersplenic–renal mass. The chief complaint was pain in the left hypochondrium that had been evolving for 1 month. Physical examination found a patient in good general condition with a 15 × 10 cm^2^ solid mass of the left hypochondrium, reaching the umbilicus, poorly limited and fixed to the deep plane. No palpable supraclavicular or inguinal lymph nodes were found. Abdominal ultrasonography showed a fairly well-circumscribed intraperitoneal mass in front of the spleen, compressing the splenic hilum with predominantly heterogeneous tissue echo structure. Eso-gastroduodenal endoscopy showed extrinsic compression of the anterior face of the gastric body. Colonoscopy was normal. Abdominopelvic computed tomography (CT) showed an intraperitoneal tissular mass infiltrating the left abdominal wall, extending from the left diaphragmatic dome to the pelvis and measuring 25 × 15 cm^2^. This mass was compressive, pushing the stomach to the right, the spleen, and the left kidney backward without invading them. The pancreas seemed to be taken by the mass. The patient underwent an exploratory laparotomy, finding a large, firm, hypervascular, extended mass with multiple nodules of carcinomatosis. Biopsy confirmed the malignancy of the lesion as well as nodules. Due to the extent of the disease, the tumor was considered unresectable.

Histopathological and immunohistochemical studies revealed DSRCT. The patient underwent one cycle of chemotherapy based on vincristine, ifosfamide, doxorubicin, etoposide (VIDE). The patient did not tolerate well this chemotherapy. He had hospitalization for febrile neutropenia.

The patient died 5 months after the diagnosis.

### Case 2

A 17-year-old North African male patient with no past medical history was referred by his doctor to our department to manage a pelvic mass. The chief complaint was hypogastric pain and dysuria that had been evolving for 2 months. Physical examination found a patient in good general condition with a firm hypogastric 20-cm-long axis mass, multilobular, fixed, and extending up to the umbilicus, associated with 2 cm right inguinal lymphadenopathy. On digital rectal examination, the tumor’s lower pole was palpated infiltrating prostate. Abdominopelvic CT showed retrobladder mass inseparable from the prostatic base, infiltrating the bladder floor associated with prerectal and paraaortic lymphadenopathy. The patient underwent surgery. Peroperatively, the presence of a pelvic tumor measuring 20 × 15 cm^2^ was found, intimately associated with bladder and rectum, with multiple nodules along the iliac axes and on the omentum. We performed debulking surgery with persistence of tumor residue. Histopathological and immunohistochemical studies revealed DSRCT. The patient underwent chemotherapy based on vinblastine and methotrexate with 60 Gy pelvic radiation therapy, with disappearance of tumor residue. The evolution was marked by a relapse 3 months after end of chemotherapy and the appearance of metastatic liver lesions and cœliomesenteric lymphadenopathy on control CT. The patient underwent chemotherapy based on VIDE without remission. We lost him after 5 years of follow-up.

### Case 3

**Fig. 1:**
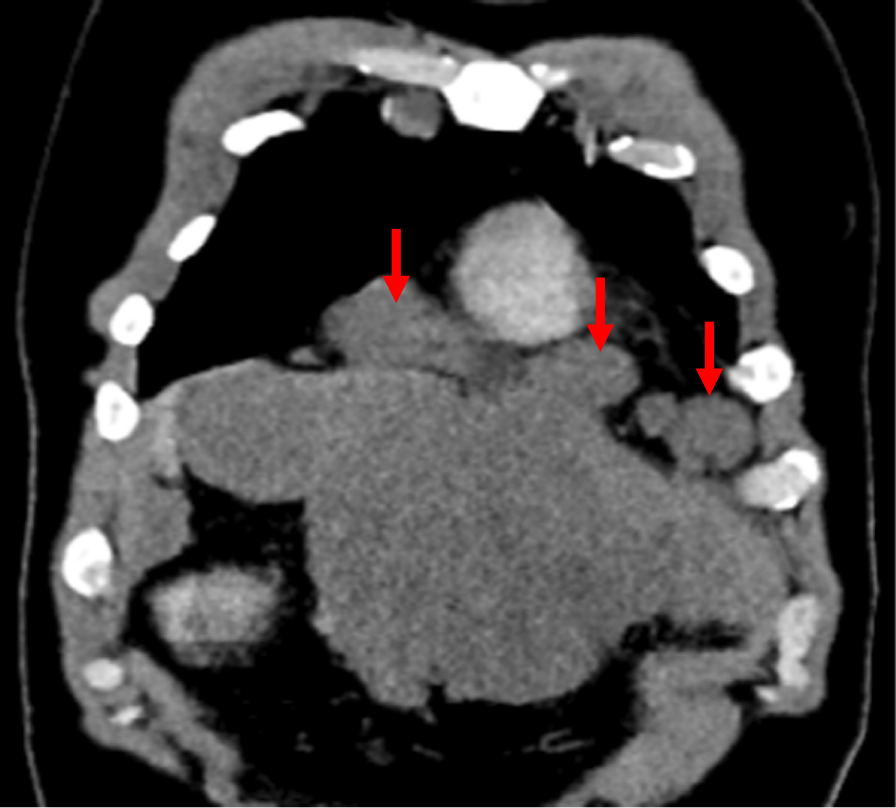
Case 3: coronal section of thoracoabdominal CT scan, showing multiple bilateral mediastinal adenopathies of anterior cardiophrenic angles (red arrows)

**Fig. 2:**
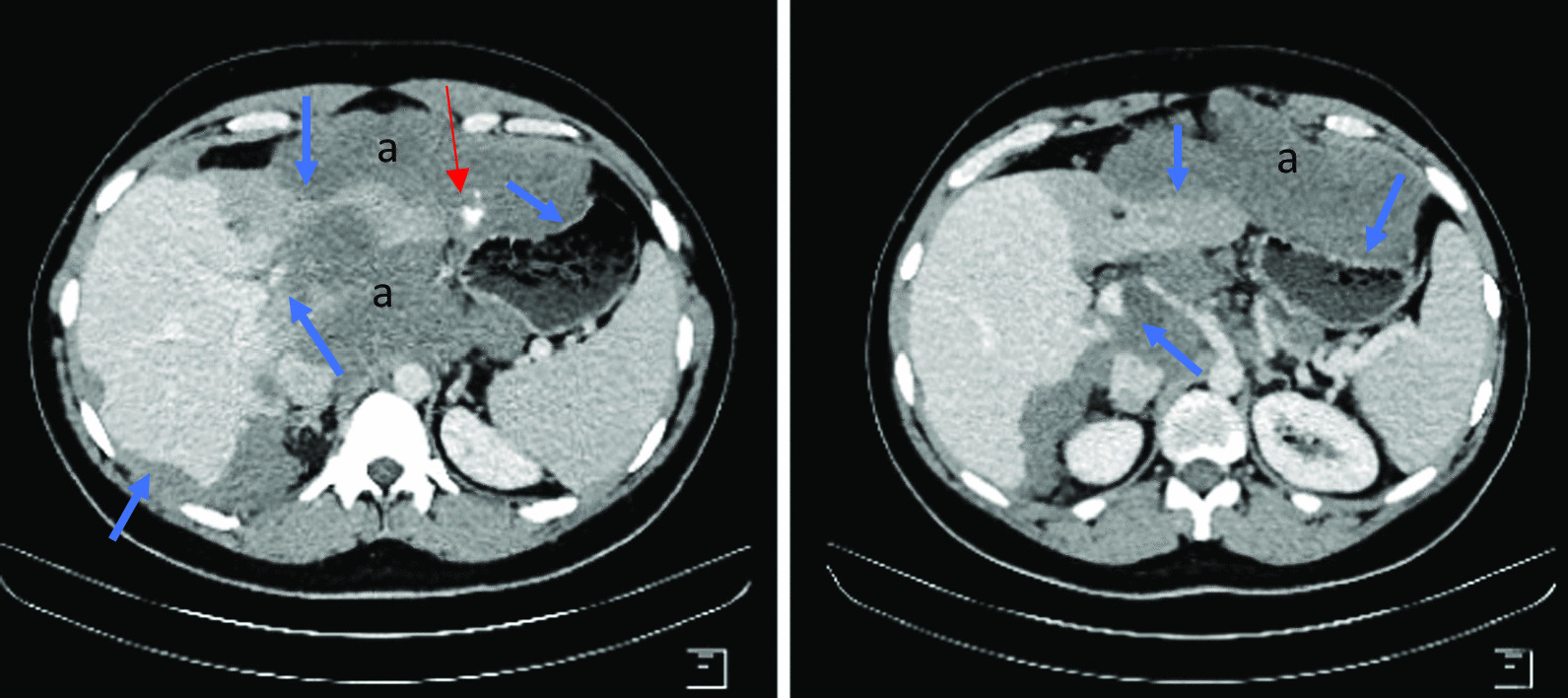
Case 3: axial sections of abdominal CT scan with injection at venous time, showing tissue mass (**a**) responsible for a mass effect on the stomach and scalloping on the left liver, hepatic hilum, and perihepatically (blue arrows). Red arrow indicates some calcifications inside the tumor

**Fig. 3:**
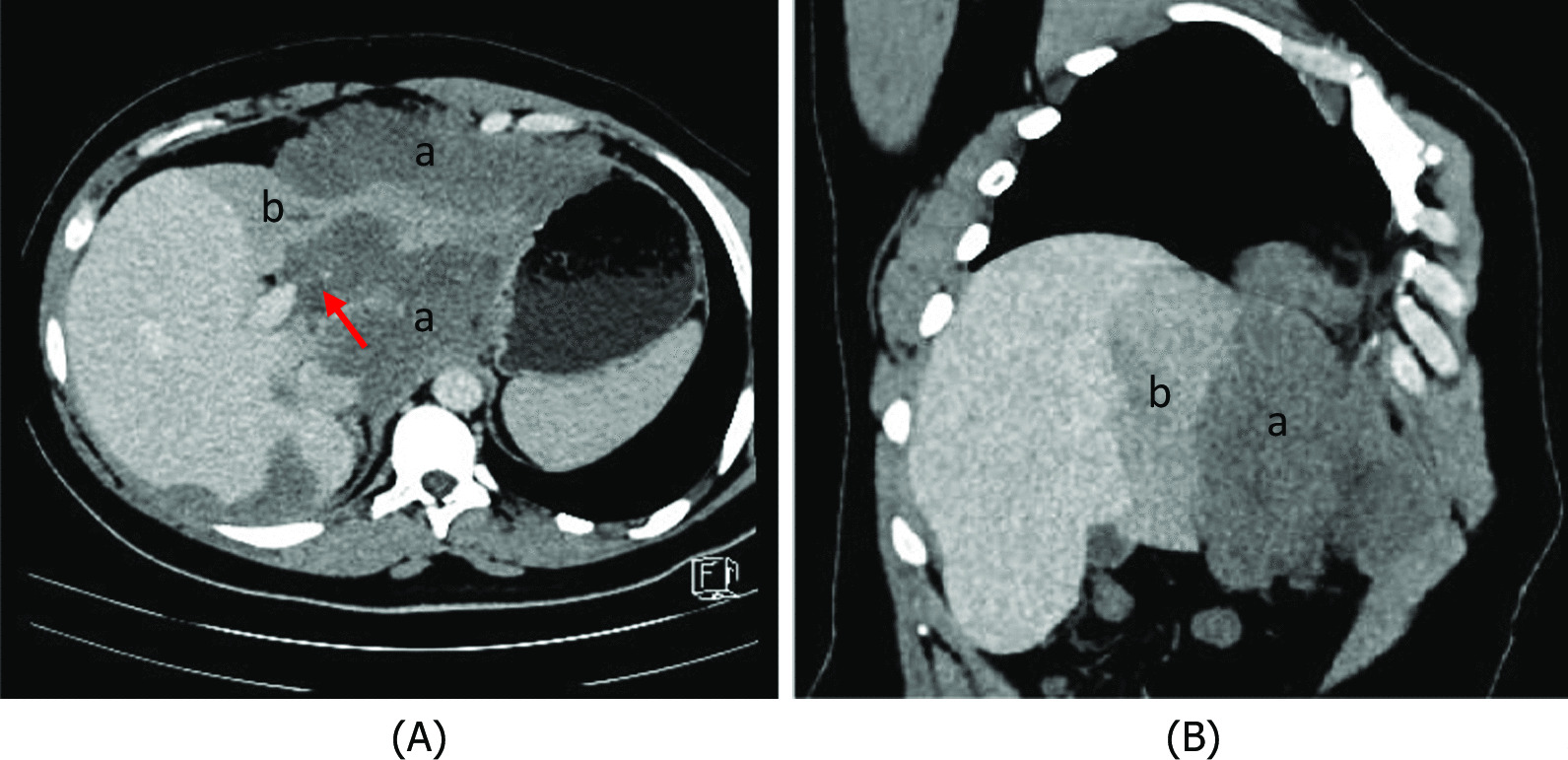
Case 3: axial (**A**) and coronal (**B**) sections of abdominal CT scan with injection at venous time, showing tissue mass (**a**) responsible for scalloping on the left liver and hepatic hilum with obstruction of the left portal branch (red arrow) causing hypoperfusion of the left liver (**b**)

**Fig. 4:**
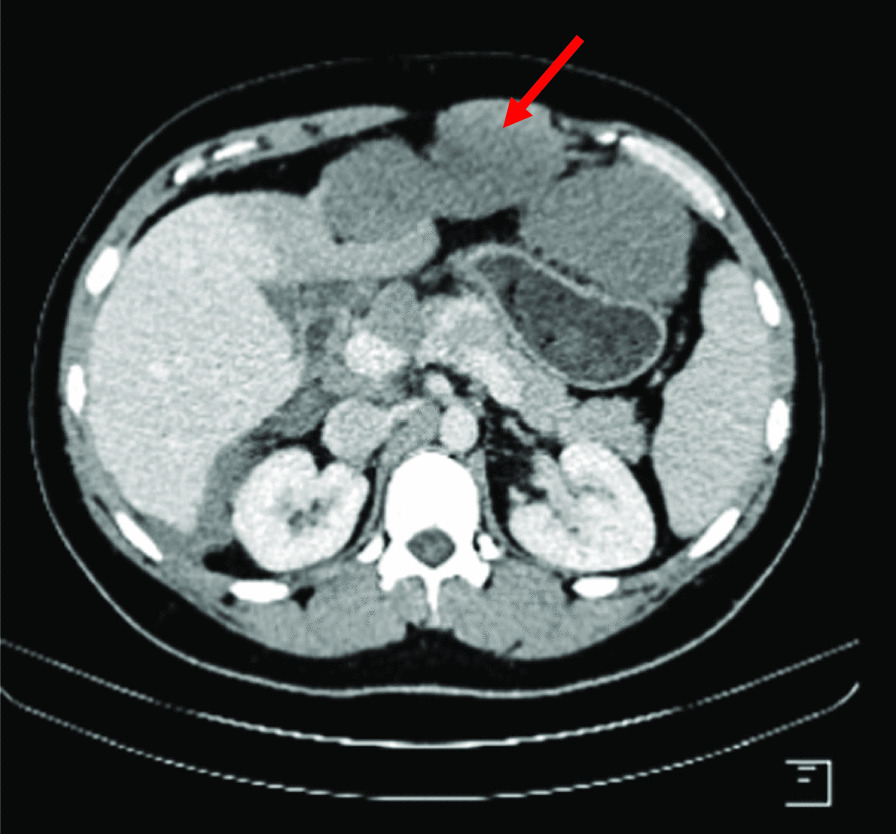
Case 3: axial section of abdominal CT scan with injection at venous time, showing invasion of the left rectus abdominis muscle by the tumor (red arrow)

A 30-year-old North African patient with no notable pathological history consulted for management of an intraabdominal mass. The patient complained of epigastralgia and gastroduodenal reflux for 3 months leading him to take quadruple therapy for *Helicobacter pylori* but without improvement. Physical examination found a patient in good general condition, with a 20-cm-long axis firm epigastric mass, fixed and multilobular without palpable lymphadenopathy. Abdominal ultrasonography showed a poorly limited hypoechoic mass in the perigastric area. Eso-gastroduodenal endoscopy showed extrinsic compression of the anterior face of the gastric body. Thoracic-abdominopelvic CT scan showed:

- In the thoracic stage: Right phrenic angle mass of 48 × 21 mm^2^ associated with mediastinal adenopathies (Fig. 1).
- In the abdominal stage: Tissue mass, with calcifications, centered on the small omentum with extensions to the left liver. It was responsible for a mass effect on the stomach by coming into contact with the cardia, the small curvature, and the rectus abdominis muscle of the abdomen, measuring 210 × 190 × 130 mm^3^. This mass was also responsible for scalloping of the liver and the hepatic hilum with obstruction of the left portal branch, causing hypoperfusion of the left liver (Figs. 2,3,4)

Presence of multiple other intraperitoneal masses was noted, especially in the perihepatic, upper mesenteric, interaortic cave, and at the level of the diaphragmatic plates.

**Fig. 5:**
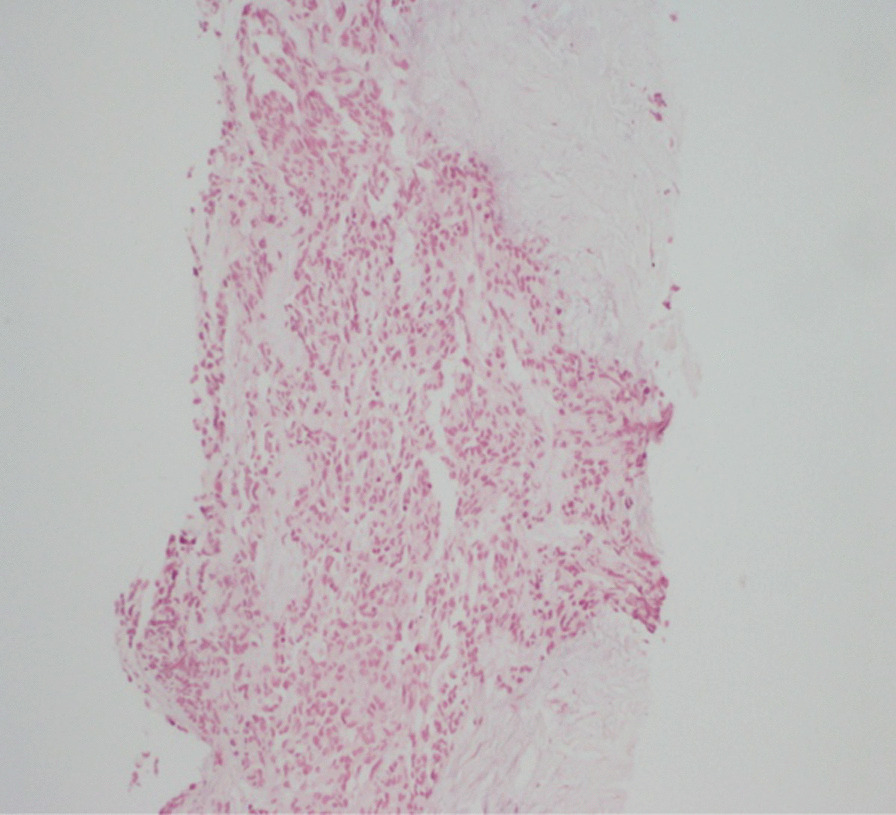
Case 3: hematoxylin and eosin (H&E) staining, showing proliferation with round cells arranged in clusters and spans in a moderately abundant fibrous stroma

**Fig. 6:**
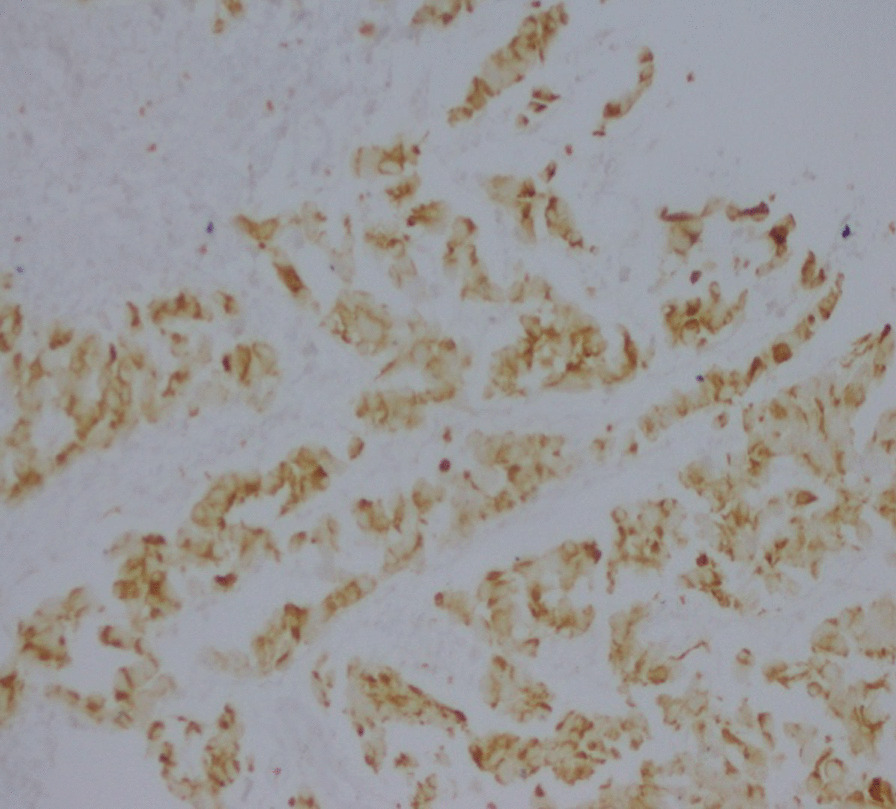
Case 3: immunohistochemistry (IHC), showing diffuse positivity with desmin

Biopsy of the epigastric mass was performed. Histopathological and immunohistochemical studies revealed DSRCT: CK (−); desmin (+); myogenin (−); chromogranin (−); synaptophysin, c-KIT (−); Dog 1 (−); CD34 (−); WT1 (−) (Figs. 5,6).

The patient underwent five cycles of chemotherapy based on VIDE with partial response. The patient had debulking surgery. We performed partial resection of the oblique muscle, peritoneum, and pericardium, resection of segment II of the liver, and omentectomy.

Postoperatively, the patient underwent thoracoabdominal volumetric modulated arc therapy (VAMT) radiotherapy and adjuvant chemotherapy based on cyclophosphamide. He is still alive after 1 year of follow-up.

### Case 4

A 16-year-old North African male patient with no past medical history consulted for diffuse abdominal pain with anorexia and weight loss of 10 kg in 1 month. Physical examination found him to be afebrile with tenderness of the whole abdomen with no signs of peritonitis. We noted the presence of a 2 cm left supraclavicular lymph node associated with a left inguinal lymph node. Abdominopelvic CT showed suspect cœlio-mesenteric and paraaortic lymph nodes with no other abnormality. We performed biopsy of the supraclavicular lymph node. Histopathological and immunohistochemical studies revealed DSRCT. Considering the absence of a tissue mass, we reached the diagnosis of ganglionic DSRCT. We referred the patient for chemotherapy. He underwent six cycles of VIDE chemotherapy with no clinical response. We tried second-line chemotherapy based on vepeside, ifosfamide, cisplatin (VIP), with no remission. The patient was lost after 2 years of follow-up.

## Discussion

DSRCT is a rare soft tissue malignancy of mesenchymal cell origin. It is characterized by poor prognosis with 15% overall survival at 5 years [4]. Young male patients are the most affected, with mean age at presentation of 22 years [5]. Tumor site is most frequently in the abdomen or pelvis. One can also find lesions in the thoracic cavity or testicle, with local or distant lymph node involvement. In case 4, we report exclusive ganglionic DSRCT with no tissue mass.

Presence of translocation t(11:22) (p13:q12), which results in active fusion of Ewing sarcoma (EWS) and Wilms tumor (WT1) genes, confirms the diagnosis of DSRCT [2].

The usual presenting symptoms are abdominal distension and discomfort, or also abdominal pain and constipation. Typically, as in our first three cases, patients present at diagnosis with large masses and/or extensive seeding in the peritoneum. Symptoms appear when peritoneal surfaces are infiltrated with tumor, overwhelming the peritoneum and impairing resorption of peritoneal fluid, causing ascites.

Due to the diffuse serosal spread of DSRCT, Hayes-Jordan and colleagues at MD Anderson Cancer Center, Houston, TX [5] established a new staging system:

- Stage 1: patients with limited disease, localized to one or two sites in the abdomen or one site elsewhere
- Stage 2: patients with any amount of extensive peritoneal disease
- Stage 3: patients with liver metastasis and peritoneal disease
- Stage 4: including peritoneal and liver disease as well as disease outside the abdominal cavity and lymph nodes.

This proposed staging system has not yet been validated. Imaging may play a central role in diagnosis of DSRCT. Computerized tomography (CT) is the most useful initial imaging study, and magnetic resonance imaging (MRI) can be helpful for pelvic and hepatic lesions. DSRCT has no specific imaging characteristics. Multiple peritoneal implants can be seen on both CT and MRI, which increases suspicion of this disease. Moreover, positron emission tomography (PET) imaging may be used to evaluate disease extension at presentation and monitor response to therapy [6–8]. Treatment of DSRCT is essentially based on chemotherapy, debulking surgery (with a goal of at least 90% reduction of tumor bulk), and radiation therapy, albeit without clear consensus [9, 10]. We performed this treatment sequence on the patient in case 3.

Despite all these multimodality therapies, recurrence seems to be the rule. For some authors, high-dose chemotherapy associated with extensive surgery, tumor debulking, with or without whole-abdominal radiation therapy can control the disease and slightly improve long-term survival [9, 11, 12]. Intensive alkylator-based chemotherapy consists of a combination of cyclophosphamide, doxorubicin, and vincristine alternating with ifosfamide and etoposide [10]. This regimen can be quite toxic, and febrile neutropenia is frequent. However, Aguilera *et al*. reported an alternative, more tolerable treatment. This is an outpatient regimen based on neoadjuvant vincristine, ifosfamide, dexrazoxane/doxorubicin, and etoposide. This treatment is followed by aggressive surgical resection and adjuvant radiotherapy. This regimen yielded a disease-free interval of approximately 2 years, providing good quality of life with regular school attendance and participation in planned activities [13]. Local control is essential in DSRCT. Aggressive cytoreductive surgery plays a primary role in local control and the achievement of prolonged survival of some malignancies involving the peritoneum [14–16]. In particular, surgical experience with DSRCT is essential to achieve complete cytoreduction (CC0, R0), which should be the preferable goal for surgery in these patients [10]. Lal *et al.* reported 3-year overall survival (OS) of 58% with gross total resection compared with 0% in the non-resection cohort [4]. In his series, Saab *et al.* reported poor outcomes for patients with abdominopelvic DRSCT in which total surgical resection was not feasible [17]. Even after chemotherapeutic cytoreduction and surgical resection, microscopic residual disease may persist in most cases. In this context, hyperthermic intraperitoneal chemotherapy (HIPEC) has been examined as a a potentially effective adjunctive intraoperative strategy in patients with DSRCT [18–20]. Recently, in a phase 2 clinical trial, Hayes-Jordan *et al.* observed 3-year overall survival of 79% when adding HIPEC to cytoreductive surgery [21]. For Honoré *et al.*, the influence of HIPEC on OS and disease-free survival (DFS) was not statistically conclusive [20].

Current research is focused on developing targeted immunotherapy using monoclonal antibodies directed at DSRCT antigens [22, 23]. Two DSRCT cell surface antigens have been identified: GD2, recognized by the antibody 3F8, and the immunomodulatory molecule B7H3, recognized by the antibody 8H9 [24, 25]. Several other potential therapeutic targets for DSRCT are currently under development [22]. This new therapeutic approach may have better tolerability and may decrease toxicity because these antibodies specifically target antigen-expressing neoplastic cells and have restricted cross-reactivity with most normal tissues [26, 27].

## Conclusions

DSRCT remains a lethal disease, affecting primarily males in adolescence and young adulthood. Current treatment prolongs life but rarely achieves cure. Tumor debulking, chemotherapy, and radiotherapy has been shown to prolong survival. New therapeutic approaches, such as HIPEC and intraperitoneal antibody instillation, are being studied. Reporting of more case series is crucial to improve management of this pathology.

## Data Availability

We took data supporting our findings from the patients’ records.
